# Goose management schemes to resolve conflicts with agriculture: Theory, practice and effects

**DOI:** 10.1007/s13280-016-0884-4

**Published:** 2017-02-18

**Authors:** Einar Eythórsson, Ingunn M. Tombre, Jesper Madsen

**Affiliations:** 1 High North Department, Fram Centre, Norwegian Institute for Cultural Heritage Research (NIKU), P.O. Box 6606, Langnes, 9296 Tromsø, Norway; 2 Department of Arctic Ecology, The Fram Centre, Norwegian Institute for Nature Research (NINA), P.O. Box 6606, Langnes, 9296 Tromsø, Norway; 3 Aarhus University, Rønde, Denmark

**Keywords:** Agriculture, Pink-footed goose, Refuge areas, Wildlife management

## Abstract

In 2012, the four countries hosting the Svalbard population of pink-footed goose *Anser brachyrhynchus* along its flyway launched an International Species Management Plan for the population. One of the aims was to reduce conflicts between geese and agriculture to an acceptable level. Since 2006, Norway has offered subsidies to farmers that provide refuge areas for geese on their land. We evaluate the mid-Norwegian goose management subsidy scheme, with a view to its adjustment to prevailing ecological and socio-economic parameters. The analysis indicates that the legitimacy of the scheme is highly dependent on transparency of knowledge management and accountability of management scheme to the farming community. Among farmers, as well as front-line officials, outcomes of prioritisation processes within the scheme are judged unfair when there is an evident mismatch between payments and genuine damage. We suggest how the scheme can be made more fair and responsive to ecological changes, within a framework of adaptive management.

## Introduction

Conflicts between wildlife and local human interests are significant in many parts of the world (Patterson 1991; Redpath et al. 2013; Tveraa et al. 2014; Shackelford et al. 2015). In some cases, the introduction of subsidies and safe refuges for wildlife reduces the conflicts (Cope et al. 2005; Tombre et al. 2013; Madsen et al. 2014), but not in others (Bearzi et al. 2011; Besnard and Secondi 2014; Tveraa et al. 2014).

The high-Arctic Svalbard-breeding population of the pink-footed goose *Anser brachyrhynchus* has successfully adapted to landscapes modified by intensive agriculture outside the breeding area (Van Roomen and Madsen 1992; Fox et al. 2005; Chudzinska et al. 2015). The pink-footed geese stay in Belgium, the Netherlands and Denmark during winter and stage in Norway on their migration towards and from their breeding grounds in Svalbard. During the last three decades, the population has tripled, from 25 000 in 1984 to around 75 000 in 2014 (Madsen et al. 2015). Pink-footed geese make use of two major spring staging areas in Norway: Vesterålen in Nordland, where they solely forage on pastures (Tombre et al. 2010), and Nord-Trøndelag, where they forage on a mixture of stubble fields, pastures and newly sown cereal fields (Madsen et al. 1997; Madsen 2001; Tombre et al. 2008a, b; Chudzinska et al. 2015). Foraging on waste grain in stubble fields causes no problems for agriculture, but spring foraging on new growth grass causes substantial yield reduction (Bjerke et al. 2013).

Following prolonged conflicts between farmers and environmental authorities in Norway, a subsidy scheme for farms affected by spring staging migratory pink-footed geese and barnacle geese *Branta leucopsis* was introduced in 2006 in two counties; Nordland in north Norway and Nord-Trøndelag in mid-Norway. The aims of the scheme were ecological (accommodating geese) and socio-economic (reducing the conflict between geese and agriculture). The scheme was established as a result of a conflict between wildlife and agriculture; its official aims have a character of a compromise between a wildlife approach and a farmer approach. *The wildlife approach* establishes the objective to set aside sufficient refuges for the geese, within their preferred areas, while variability in goose density within the refuges is not considered a problem. *The farmer approach* is that subsidies should represent a reasonable compensation for goose refuges on their land, directly linked to actual harvest loss from grazing.

According to the policy documents prepared by the Ministry of Agriculture in 2006, the primary goal of the scheme was to secure refuges for pink-footed geese and barnacle geese in the spring staging areas. Compensation for harvest loss was not directly mentioned, subsidies were defined as payments for securing refuge areas in order to maintain a viable and sustainable population. The practical implementation of the scheme was handed over to the county governors’ agricultural departments, which were to decide what kind of information was needed, how to prioritise refuge areas and how to decide suitable subsidy rates for refuges (Ministry of Agriculture and Food 2006).

Within the framework of the African-Eurasian Waterbird Agreement (AEWA), the four countries hosting the Svalbard population of pink-footed goose along its flyway launched an International Species Management Plan (ISMP) in 2012. The goals of the plan were (1) to maintain a sustainable and stable population within its range, (2) keep agricultural conflicts to an acceptable level, (3) avoid enhancing degradation of tundra vegetation in the breeding range and (4) allow for recreational hunting that does not jeopardise the population. One of the plan’s objectives was to support the evaluation and optimisation of national and regional compensation/subsidy schemes and alternative non-consumptive methods to minimise agricultural conflicts in the range countries (Madsen and Williams 2012; Madsen et al. 2017). The ISMP is based on an adaptive management framework, understood as an approach for simultaneously managing and learning from implementation of management measures. Adaptive management implies incorporating scientific research into the overall management scheme and continual monitoring of ecological and socio-economic variables. Management measures are regularly evaluated and adjusted to observed changes in these variables (Williams 2011). Adaptive management is more than a scientific approach to environmental management, as it is based on social and institutional learning and deals with the unpredictable interactions between people and ecosystems as they evolve together (Berkes and Folke 1998).

The Norwegian subsidy scheme is an important management measure to reach the ISMPs goal to keep agricultural conflicts to an acceptable level. For the purpose of evaluation, the goal of the subsidy scheme is important. As the scheme was established in response to a conflict, conflict resolution is an underlying premise, as underlined in the second goal for the ISMP. Creation of refuges to accommodate geese is also an obvious goal. How this should be practiced in the implementation phase is however not clear from the policy documents. The scheme can thus not be evaluated in relation to one clear cut goal; there are different interrelated goals as well as different approaches to implementation of these goals: (1) Accommodation of geese to secure viable and sustainable population; (2) Alleviation of conflict between wildlife and agriculture. An implicit third goal, compensation for crop damage from goose grazing, is not stated outright in the policy documents.

For the evaluation of the subsidy scheme in Nord-Trøndelag (2006–2015), we have applied criteria developed by Rauschmayer et al. (2009), namely *knowledge management, social dynamics, legitimacy and effectiveness*. These criteria have been applied to the evaluation of policy outcomes and policy processes in European governance of natural resources. We outline the initial design of the scheme, how it has been revised and expanded. We further examine the relationship between the growth of the pink-footed goose population and the amount of subsidies allocated from the scheme. We also show how allocation of funds from the scheme is related to social dynamics between farmers and agricultural agencies, and how management of measurement costs strikes a balance between legitimacy and effectivity. Finally, we suggest how the scheme, and its implementation, can be made more responsive to ecological changes, within a framework of adaptive management.

## Materials and methods

The information presented is based on interviews with farmers and government officials. Numerical data on distribution of subsidies and refuge areas in 2006–2015 were kindly provided by the Nord-Trøndelag County Governor’s administration. Biological data on population status and migration of pink-footed goose were provided by the Norwegian Institute for Nature Research (NINA) and Aarhus University. These data were compiled by two research projects; *MIGRAPOP* (Eythórsson and Tombre 2013; Tombre et al. 2013) and *GEESE BEYOND BORDERS.*

**Fig. 1:**
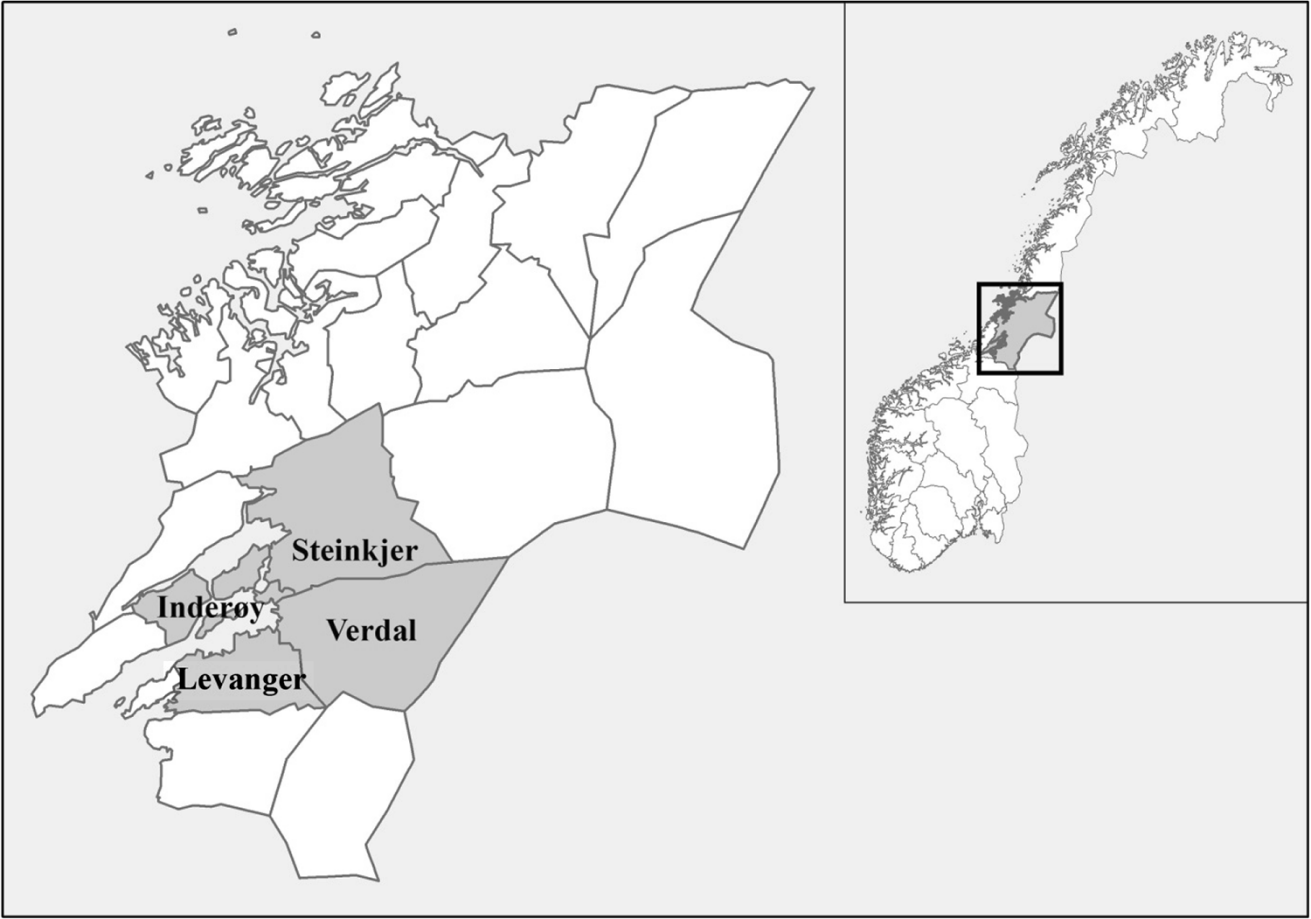
Location map of Nord-Trøndelag (*insert*), the main spring and autumn staging area of pink-footed goose in Norway, covered by the municipalities of Steinkjer, Inderøy, Levanger and Verdal (Levanger and Verdal named as Inherred samkommune)

Since 1980, international coordinated goose counts, including those at the Norwegian stopover areas, have been carried out in order to estimate total population size (for details see Madsen et al. 2014, 2015). Since more than 90% of the pink-footed goose population is concentrated in Nord-Trøndelag (shown in Fig. 1) from late April to mid-May (e.g. Madsen et al. 2015), we use the total population size as an expression of the goose numbers present.

In 2011, we interviewed local officials (*n* = 4) responsible for administrating the subsidy scheme in three municipalities: Steinkjer, Innherred samkommune and Inderøy (see Fig. 1). Approximately, 90% of the subsidies allocated to farms in Nord-Trøndelag 2006–2015 were distributed within these municipalities. We also interviewed the regional officials (*n* = 4) responsible for the subsidy scheme in the County Governor’s agricultural administration in 2011, 2012 and 2015. Interviews with local and regional officials focused on the subsidy scheme; prioritisation practices, experiences from administrating the scheme and responses from farmers.

Eight farmers were interviewed in 2011, four of whom were participating in the subsidy scheme, while the other four had not applied for subsidies. In the interviews with farmers, the focus was on the total impact of geese on the farm, long-term effects of goose grazing and how the subsidy scheme worked from the farmers’ perspectives. The farmers were selected considering the geographical distribution and location of farm within the goose area. Obviously, a sample of eight farmers is too small to be representative for all farmers in the region, but these interviews were combined with interviews with eight agricultural officials who receive responses from all farmers within the subsidy scheme.

## Results

### The subsidy scheme and its implementation

The subsidy scheme was based on payment in advance to farmers who offer some of their land as a goose refuge. There was an understanding that goose refuges can only be established on land with high density of geese. The details of the scheme and its implementation were devolved to the regional agricultural administration. From 2006 to 2013, the agricultural administration in Nord-Trøndelag chose to apply one flat subsidy rate, which did not distinguish between heavily grazed and moderately grazed land.

Interviews with farmers and employees of the agricultural administration in Nord-Trøndelag revealed that while the regional branch of the Farmers’ Union was engaged in the goose issue on the regional level, responses from farmers about the subsidy scheme were usually in the form of direct interactions between individual farmers and local agricultural officials.

The biological outcomes of the subsidy scheme, in terms of goose accommodation on subsidised fields in Nord-Trøndelag, have been evaluated for the spring seasons in 2007, 2008 and 2010 (Tombre et al. 2008b, 2009; Madsen et al. 2014). Tombre et al. (2009) noted that in 2008, only a small fraction of the geese was observed within the refuge areas. In order to improve the predictions of goose distribution, and thus get a better spatial match between grazing pressure and refuge areas, they recommended the use of a species distribution model, developed in a research project funded by The Research Council of Norway (RCN). This model provided a spatially explicit prioritisation tool for the subsidy scheme (Jensen et al. 2008). Before the spring season in 2009, a meeting was organised at The County Governor in Nord-Trøndelag where the researchers presented the model that predicts goose distribution, and proposed principles for its’ implementation as a part of the subsidy scheme. The agricultural administration decided to use the model and during 2009–2013, applications for subsidies in Nord-Trøndelag were prioritised according to this model. The model predicted which pastures were likely to be preferred by geese and ranked pastures according to suitability of the landscape for foraging geese; primarily identified by proximity to roads and vertical structures, distance to roost sites on the coast, lakes or larger rivers and elevation above sea level (Jensen et al. 2008). Based on data from 2009 and 2010, the outcomes from the use of the model as a prioritisation tool for ranking of pastures were evaluated (Madsen et al. 2014). As a measure of goose usage, density of goose droppings was compared inside and outside subsidised refuges at the end of the 2010 foraging season; the results showed that goose grazing pressure was 13 times higher on subsidised pastures compared to a stratified random selection of non-subsidised pastures. It was also calculated that 67% of goose grazing pressure on grassland in Nord-Trøndelag in 2010 was exerted on refuges, although the refuges only comprised 13% of the total pasture area available. Madsen et al. (2014) concluded that the outcomes of the subsidy scheme, in terms of spatial match between foraging areas and refuges, clearly showed improvements from 2008 to 2010, thanks to the use of the species distribution model. Consequently, the scheme had become more effective, relative to the biological objective of accommodating geese on subsidised refuges as well as in terms of the cost-effectiveness of the distribution of subsidies. It was also recognised that regular updates would be needed to incorporate new pastures when geese changed their foraging pattern/site use or densities increased as a consequence of changed distribution and continued population growth.

Development and updating of the model represent an investment of considerable effort and costs which were covered by research grants. The original version of the model was based on goose distribution and abundance data collected over the period 2004–2007. This version was applied as a basis for processing subsidy applications from farmers from 2009 until 2013. Most of the measurement costs represented by the model were covered by RCN project funding. Future updates could thus not be guaranteed, as updating was dependent on continued project funding. As a part of a new RCN-funded research project, an updated version of the model was developed in 2011–2012, based on more detailed maps and updated goose distribution data from extensive registrations of goose droppings (Simonsen 2014; Baveco et al. 2017). The revised model was used as a basis for the distribution of subsidies in 2014. In 2015, however, the agricultural administration of Nord-Trøndelag decided to carry out their own annual evaluations of crop damage on affected pastures, rather than relying on the species distribution model. It was decided that payments would be based on registration of previous year’s crop damage reported by the farmers and verified by municipal agricultural officials in terms of grass height measurements. Hence, this new practice has moved the subsidy scheme from the advance provision of money for securing refuge areas for geese in the direction of post hoc compensation to farmers. Annual verification of crop damage is likely to generate substantial measurement costs which have to be covered by the agricultural administration.

### Responses from farmers and administrators

The farmers who were interviewed in 2011 all agreed that the subsidy scheme had alleviated the conflict between geese and agriculture. Nevertheless, they were critical of the distribution of subsidies for not reflecting the actual harvest loss experienced by individual farmers. Payments in advance were also criticised for missing the target because of the spatial variability in goose distribution from one year to the next. There were different opinions of whether the subsidy rate was sufficient to cover real losses. One farmer noted that the flat subsidy rate was too low for heavily grazed areas. Farmers who do not have refuges on their land are free to scare geese away, but one farmer pointed out that in the long run, scaring geese was a poor alternative to subsidies, even if the payments were insufficient relative to harvest loss.

Local officials in the agricultural administration interviewed in 2011 all agreed that the practice of payments in advance was perceived unfair by the farmers. The officials argued that the scheme had made life easier for the geese, but it did not reflect the harvest loss for individual farms. In their opinion, payment in advance was problematic because of variable grazing patterns; post hoc compensation would match the actual losses more accurately and would meet greater understanding from farmers.

In an interview in 2011, the head of the regional agricultural administration explained that the use of the species distribution model as a basis for subsidy allocation was very useful for the municipalities, since they could use it to legitimate their allocation of subsidies; they could refer to the model to show that decisions were not arbitrary.

In 2015, the regional administration assessed the species distribution model differently. The County Governor had, after discussions with the affected municipalities and the Farmers’ Union, decided no longer to base the allocation of subsidies on the model. It was also decided to apply two subsidy rates, differentiated according to the severity of crop damage, instead of the previous flat rate. The municipalities had been uncomfortable with the poor match between prioritised areas suggested by the model and actual distribution of harvest loss. In answer to the question as to whether the municipalities had considered increased measurement costs that would probably arise as a result of discarding the species distribution model, the agricultural officials explained why the municipalities were willing to take the necessary expenditures to assess crop damage: “The new procedure will give them (local officials) more legitimacy, they will be able to show that the allocation is based on facts; the actual damage. Compared to the previous situation, when they were locked into the map (the model), it will be easier to say yes or no. In the other system, you might have to say; I can see that you have lost half your harvest, but your land doesn’t have the right colour on the map. Then there may be a neighbour who has the right colour, who gets subsidies, even if there is very little grazing on his land. The municipalities prefer to take on the extra work load to verify the damage, because for them, the scheme will be easier to handle” (quote from an official at the County Governor’s Agricultural Agency, March 12 2015).

### Subsidy development and goose population changes

**Fig. 2:**
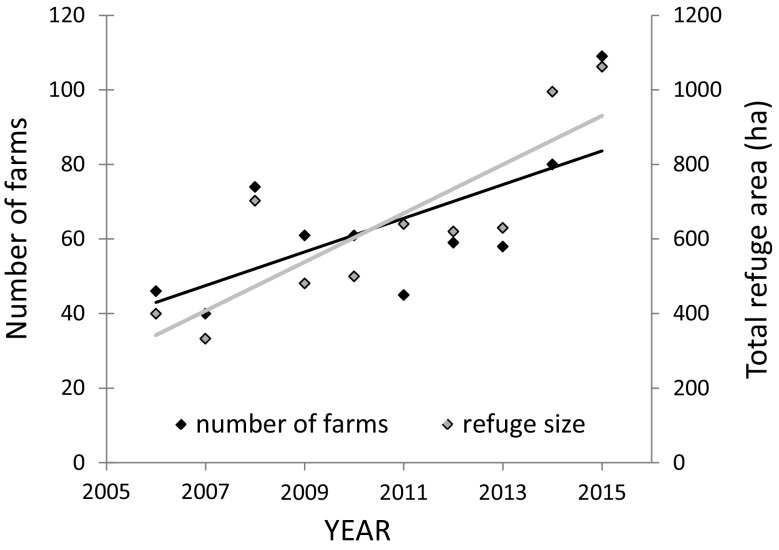
The number of farms involved in a goose subsidy scheme (*black squares*) and the total size of refuges (*grey squares*) in the county of Nord-Trøndelag, mid-Norway, over the period 2006–2015. There is a significant increase for both variables (linear regressions: number of farms (black line): *r* ^2^ = 0.45, *n* = 10, *P* = 0.033, refuge size (*grey line*): *r* ^2^ = 0.70, *n* = 10, *P* = 0.003)

**Fig. 3:**
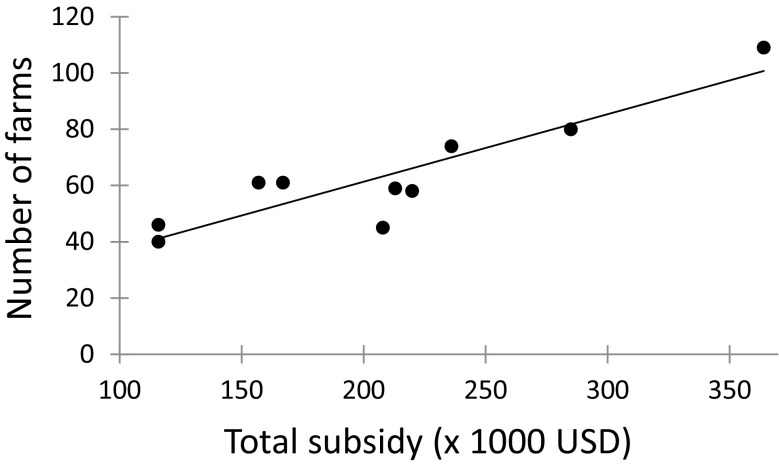
The positive relationship between the number of farms in a subsidy scheme in the county of Nord-Trøndelag in mid-Norway over the period 2006–2015 and the total amount of subsidies each year (in USD) (linear regression: *r* ^2^ = 0.81, *n* = 10, *P* = 0.0004)

The refuge areas in Nord-Trøndelag have almost tripled from 2006 to 2015 and the number of farms with subsidy more than doubled (Fig. 2). Similarly, there was a significant, and positive, relationship between the number of farms included in the scheme and the total amount of money spent on subsidies, which has more than tripled over the study period (Fig. 3).

**Fig. 4:**
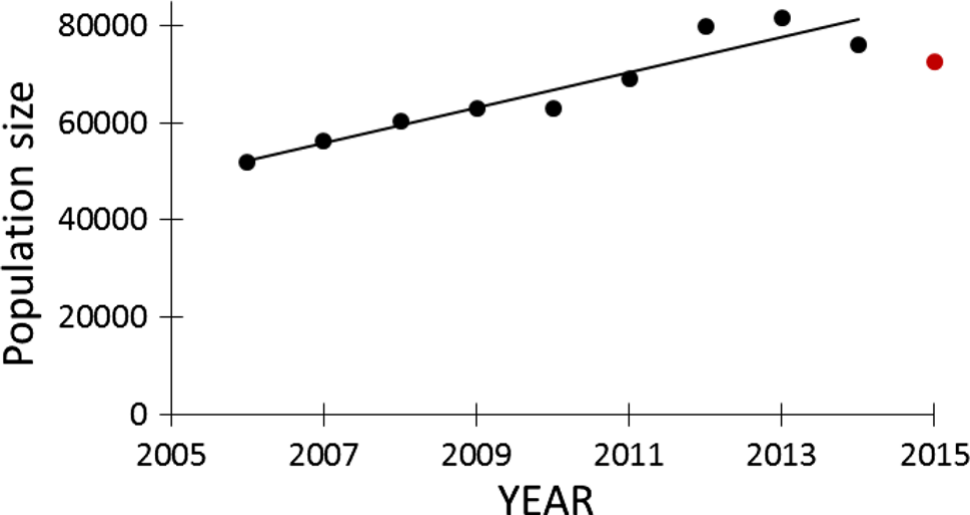
Annual increase in the size of the Svalbard-breeding population of pink-footed geese over the study period. From 2006 to 2014 the increase is significant (*r* ^2^ = 0.89, *n* = 9, *P* = 0.0001). The preliminary population estimate for 2015 is shown by the *red point*

**Fig. 5:**
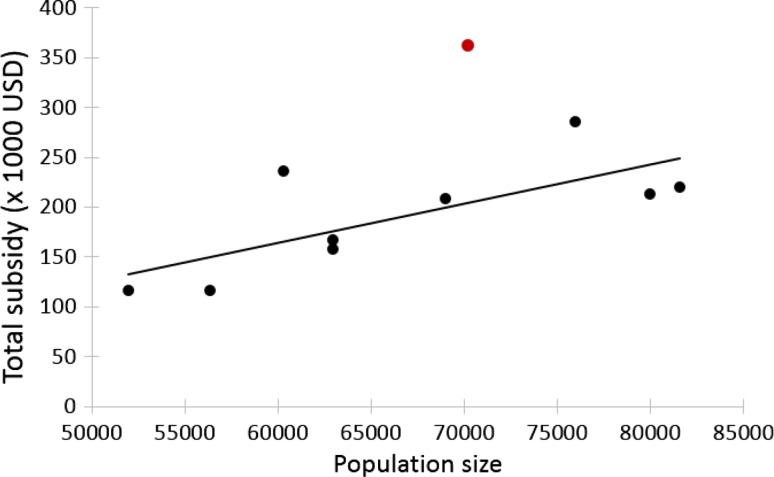
The relationship between annual total subsidies paid to farmers and the population size of pink-footed geese in Nord-Trøndelag County. The values for the year 2015 are shown by the *red point*. A significant positive relationship is only found when 2015 is excluded from the linear regression (*r* ^2^ = 0.53, *n* = 9, *P* = 0.027)

**Fig. 6:**
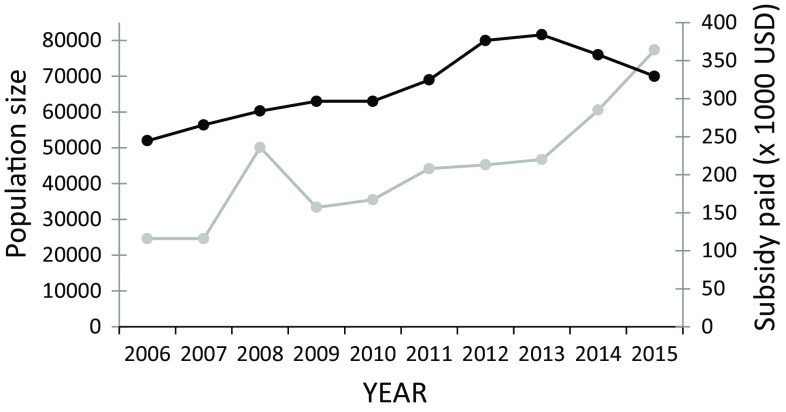
The development in the population size of pink-footed geese (*black line*) and the subsidy paid to farmers (*grey line*) in Nord-Trøndelag County in spring

During the same period, the total population of pink-footed goose increased exponentially from around 52 000 individuals in spring 2006 to 81 600 in spring 2013. In 2014 and 2015, however, the population had declined (Fig. 4), explained by an increased hunting pressure in the preceding winters. The population for 2015 is preliminary estimated to 70 000 individuals (AEWA International Working Group for the Pink-footed Goose 2015). In Fig. 5, the subsidy is plotted against the corresponding population size for the years 2006 to 2015. Excluding 2015 (the first year with a new management system in place), a linear regression analysis revealed a positive and significant relationship (*r* ^2^ = 0.53, *n* = 9, *P* = 0.027), and a relative rate of change of 1:1.5 in population size to subsidy payment, i.e. the payment has increased at 150% compared to 100% population growth, and the increase of the two variables shows a different pattern (Fig. 6).

The apparent mismatch between goose population size and funds, i.e. a steeper increase in funding than in the number of geese, may be explained by two factors. First, in the early years of the scheme, the total budget for subsidies was too small in relation to the extent of the affected area, creating a situation where farmers received significantly less funding than the real costs caused by geese. Subsequently, this may have been compensated for by increased funding. Second, political pressure by the Farmers’ Union may explain the increased funding, so the increase may reflect successful lobbying rather than the actual number of geese accommodated. In conclusion, there was a positive correlation between goose numbers and the total subsidy paid, but the steeper increase in subsidy compared to the goose population was not justified by biological data. A recent model predicting the relationship between goose numbers in Nord-Trøndelag and yield loss to grass, supports such a relationship, but with substantial between-year variation due to varying spring conditions (Baveco et al. 2017).

## Discussion

### Subsidies, prioritisation tools and their linkage to ecological parameters

The budget for subsidies to goose refuges in a given year is dependent on the result of negotiations between the Farmers’ Union and the Ministry of Agriculture for the annual Agriculture Agreement. Since 2014, the funding comes as a part of a larger national programme, the Regional Environmental Programme, for which the internal prioritisation between goose refuges and, for example, maintenance of cultural landscape/heritage, is devolved to the regional administration (County Governor of Nord-Trøndelag 2014). Changes in ecological parameters that are likely to increase or decrease harvest losses caused by geese can thus be brought into the budget negotiations between the Ministry and the Farmers’ Union. Since 2014, these parameters can also be considered in the process of deciding regional prioritisation of funds within the Regional Environmental Programme by the County Governor’s agricultural administration. The other main staging area in Norway, Vesterålen in north Norway, also hosts pink-footed goose in spring, and the distribution of geese between the two sites will influence the total pressure in Nord-Trøndelag. Climatic factors also influence the grazing pressure, as the advancement of spring over the last decade has led to earlier arrival of pink-footed goose in Nord-Trøndelag (Tombre et al. 2008a).

Once the annual budget is decided, the challenge is to distribute the subsidies in a way that meets the objectives of the scheme and is considered legitimate by the farmers, without generating excessive transaction costs in the process. In the absence of perfect knowledge of the spatial distribution of geese and the intensity of their grazing, prioritisation is based on different sets of indicators, as those derived from the species distribution model or the post hoc evaluation of grazing intensity. Daily goose counting throughout the spring staging period has also been used with some success in Nordland (Eythórsson and Tombre 2013; Tombre et al. 2013). Systematic counting of goose droppings has been used in evaluation of the subsidy scheme (Madsen et al. 2014), and as a basis for the updated species distribution model. These indicators have their strengths and weaknesses, and all generate their own measurement costs.

The data from interviews presented above explain the background for the decision by the regional agricultural administration to change the prioritising procedures and produce their own indicators by estimating crop damage post hoc, instead of relying on the species distribution model. Officials and farmers were in agreement that the model failed to reflect the reality on the ground at the detailed level on an annual basis. Local officials explained that they were embarrassed to be held accountable for decisions based on a model they could not explain, to legitimise decisions by a type of knowledge that was not immediately transparent.

Following the new procedure, subsidies will still be paid in advance, based on assessments of damage during the previous spring season. The assessments will classify affected grassland into two categories, based on two indicators; sward height at the end of the goose staging period and estimated crop reduction from goose grazing (estimated by the farmers in collaboration with local agricultural authorities). It is, as yet, too early to evaluate how these indicators will affect the distribution of subsidies, how post hoc evaluation will impact transaction costs and if it will improve the legitimacy of the scheme, compared to earlier practice based on the species distribution model. The shift from a use of flat subsidy rate and a species distribution model as a guide for prioritisation, to differentiated rates and allocation based on damage assessment can be characterised as a move away from a wildlife approach towards a farmer approach, implicitly accepting damage compensation as a goal for the subsidy scheme.

### Rauschmeyer’s criteria

Evaluation with a focus on *knowledge management, social dynamics, legitimacy and effectivity* within a governance process (Rauschmayer et al. 2009) reveals dilemmas within the subsidy scheme, which have caused tensions in the interaction between farmers and the front-line agricultural officials. These dilemmas are about how to manage knowledge within the scheme through social dynamics that exist between science, management institutions and stakeholders, in order to maintain legitimacy and effectiveness.

Knowledge management refers to the ways in which knowledge is elucidated and integrated and how the governance process addresses issues of uncertainty. In the present context, knowledge management refers to how knowledge is compiled and applied by the agricultural administration in order to estimate the spatial distribution of geese and crop damage, as a basis for allocation of subsidies to farms. Social dynamics refer to the participation of stakeholders and the degree of conflict or trust in interactions between and within government institutions and stakeholder bodies. Social dynamics in the form of stakeholder participation in decision-making about these procedures is likely to reinforce legitimacy of their outcome and to decrease the level of conflict between management institutions and stakeholders (Mitchell et al. 1997; Eythórsson 2003). Legitimacy deals with accountability, representation, rule of law and transparency. Legitimacy of process and outcome for the subsidy scheme is related to the transparency of, and trust in, knowledge management and administrative procedures. Effectiveness is a measure of policy outcomes, compared to management objectives and relative to costs. Effectiveness in the present case refers to how the outcomes of the scheme correspond to its ecological and socio-economic objectives, and whether the outcomes justify the costs. For evaluation of cost-effectiveness of damage compensation schemes, Schwerdtner and Gruber (2007) make a distinction between (direct and indirect) damage costs and transaction costs. Transaction costs, in this context, consist of measurement costs and administrative decision-making costs.

Rauschmayer’s four criteria focus on socio-economic aspects of environmental policy and refer only indirectly to ecological parameters. For example, knowledge management in natural resource governance refers to how ecological, as well as socio-economic knowledge is acquired, processed and applied. Effectiveness of a management scheme refers to attainment of ecological and socio-economic objectives.

### Knowledge management

In the absence of perfect knowledge, legitimacy of knowledge is based on consensus about measurement routines that generate workable indicators at an acceptable cost. Knowledge management is thus an exercise in balancing measurement costs against the need for credible and transparent information, which is indispensable for legitimacy of decisions taken within the subsidy scheme. If this information is imperfect and continually questioned, decision-making costs will increase. If stakeholders and governments have different priorities in terms of which goals to pursue, it also impacts their perception of what kind of knowledge is necessary and which indicators are suitable. The initial design of the subsidy scheme, as stated in national policy documents (Ministry of Agriculture and Food 2006), indicates a wildlife approach; that the goal of the subsidy scheme is to establish goose refuges to accommodate the pink-footed goose population, without detailed measurements of crop damages within individual refuges. The species distribution model, applied as a tool to identify relevant refuge areas from 2009 to 2013, was based on such approach. The decision by the regional agricultural authority to discard the model represents a shift towards a farmer approach; that the subsidies should be understood as compensation for real damage. The perceived problem with the species distribution model was not only lack of transparency, it also represented an approach that focused on refuge areas without addressing measurement of actual crop damage on individual farms.

The decision of the agricultural administration to internalise measurement costs by carrying out their own damage assessments instead of leaning on the species distribution model reduces the scheme’s dependence on input from wildlife management and research institutions. This could weaken its link to the ISMP for the Pink-footed Goose and to ecological parameters in general. Within the subsidy scheme, there is no built-in mechanism to link the amount of funding for goose refuges to population size; adjustments of subsidies to population size only take place indirectly.

### Social dynamics

Unlike EU agro-environmental schemes (Oñate et al. 2000; Kleijn and Sutherland 2003), the Norwegian subsidy scheme is governed from the bottom-up; the governance procedures used are developed regionally, in collaboration with the Farmers’ Union and local municipalities. Farmers were actively involved in the initiation of the scheme and have had an opportunity to communicate their concerns about the scheme. The number of farmers receiving subsidies is relatively small, and information about where the refuges are, and consequently about who has received subsidies, is readily available for Nord-Trøndelag (www.ntfk.no, www.gint.no). Within the farming community, a mismatch between experienced spatial distribution of damage and distribution of subsidies will be noticed and discussed among farmers and local agricultural officials. Farmers can voice problems directly to the agricultural administration or through the Farmers’ Union. For the social dynamics of the scheme, this offers an opportunity to take an adaptive approach to problem solving and addressing conflicts at an early stage. Through dialogue between stakeholders and managers, the practices have been moderated and adapted to local conditions in the two counties covered by the subsidy scheme, Nord-Trøndelag and Nordland; the two regions have developed different systems for documentation, subsidy rates and administrative procedures (Eythórsson and Tombre 2013). This also means that farmers have been able to adjust the approach of the subsidy scheme.

### Legitimacy

Interviews with farmers and local agricultural officials reveal that both groups tend to refer to the subsidies as compensation; payments that are and can only be justified by yield loss. Unjustified payments, as well as rejection of justified applications for subsidies, are therefore likely to provoke reactions. Front-line agricultural officials, who engage in direct interaction with farmers, find it difficult to maintain their own credibility in relationships with farmers, if their decisions and prioritisations are challenged as unfair and poorly justified. Outcomes that are perceived as unfair will undermine the legitimacy of the scheme, and weaken trust in relations between officials and farmers. The legitimacy of the scheme is highly dependent on transparency of knowledge management and accountability of management officials to the farming community. Among farmers, as well as front-line officials, outcomes of prioritisation processes are seen as unfair if there is a spatial mismatch between payments and real damage. This indicates that it is difficult to implement a subsidy scheme with a wildlife approach through the agricultural administration, and the need for legitimacy within the farming community leads to adjustments towards a farmer approach.

### Effectiveness

The effectiveness of the scheme relates to its goal attainment as well as cost effectiveness. In the case of the subsidy scheme, there are different interpretations of what goals are most important: accommodation of geese, alleviation of conflict or compensation for crop damage. In terms of accommodation of geese, the subsidy scheme has been evaluated as effective (Madsen et al. 2014). Among farmers and local officials, crop damage has been a focus of attention. Satisfactory compensation for yield loss on farmland is not an official goal, yet the question whether the scheme offers fair and acceptable compensation for loss to affected farms has proved crucial for its long-term legitimacy. This in turn, may be a prerequisite for attaining the other goals of the scheme. There is no quantified measure of the size of refuge areas needed to satisfy the needs of pink-footed goose or of to what extent the subsidy scheme provides value for money in terms of securing a viable and sustainable goose population. The cost of the subsidy system in Nord-Trøndelag has increased during its ten years of existence and the refuge area has tripled in size. The funds continued to increase while the pink-footed goose population declined in numbers after 2013. This could either mean that the scheme has become ‘inflated’ or that it was underfinanced from the start.

## Conclusion

Policies are usually evaluated according to whether their outcomes correspond to their goals, and if the benefits justify the costs of their implementation. In the case of the subsidy system, the goals are interrelated and some of them are not explicit. In relation to the ecological goal (accommodation of geese) the outcomes have been positively evaluated as successful results from the use of a species distribution model as a prioritisation tool by management institutions (Madsen et al. 2014). The scheme has also been evaluated positively in terms of conflict alleviation (Eythórsson and Tombre 2013). The wildlife approach, which was laid out in the 2006 policy documents, and is implicitly reflected in the species distribution model, does not consider the legitimacy issue. Insufficient transparency of knowledge management based on the species distribution model gradually weakened the legitimacy of subsidies allocation. Farmers’ sense of justice and the transparency of subsidies allocation made it difficult to defend allocations that appeared not to reflect the damage situation for individual farms. The modification of the scheme towards damage compensation is intended to improve its legitimacy and thereby alleviate conflicts between farmers and agricultural officials. Compensation for actual damage was not an explicit goal of the scheme at the outset, but after the reorientation in 2014, the focus is on the farmer approach; that allocation of subsidies must be linked to the severity of actual crop damage on individual farms. The reorientation of the scheme means that local agriculture departments are responsible for damage assessment, and have to cover the increased measurement costs of evaluating crop damage on each affected farm.

The subsidy scheme is an example of bottom-up governance; it was established as a result from local initiatives and its implementation is devolved to regional and local authorities. The scheme has few links to the ISMP or Norwegian wildlife management; there is no mechanism that links the funding of subsidies to the development of the pink-footed goose population. In an adaptive perspective, linking the top-down management represented by the ISMP and the bottom-up governance represented by the subsidy scheme requires social and institutional learning on both sides. Better co-ordination between the two could be enhanced by closer involvement of the agricultural administration in the ISMP process. The weight of ecological parameters in decision-making within the agricultural administration might also be increased by providing annual science updates for participants in budget discussions about funding of the subsidy scheme. To achieve this, it is necessary that the agricultural authorities openly publish the measurements of damage, so that all parties can learn from the process and actions taken.
